# Supplementary material for: Mutation Analysis of SARS-CoV-2 Variants Isolated from Symptomatic Cases from Andhra Pradesh, India
Source: Viruses. 2023 Jul 29;15(8):1656. doi: 10.3390/v15081656 (PMC10458099; doi:10.3390/v15081656)
Supplement: Supplementary file 1 [file viruses-15-01656-s001.zip › viruses-2481969-supplementary.pdf]

Supplementary Figures and Table

Due to their large size, the images and GIF videos are converted to pdf files. High-quality images
(supplementary Figures S1, S7, S8, and S9) and video files in GIF format (supplementary Figures S2–S6; 3D structure of Spike protein with mutations labeled) and master table can be downloaded from the below link. https://zenodo.org/search?page=1&size=20&q=8046107
Title: supplementary figures for the manuscript titled "Mutation Analysis of SARS CoV2 Variants Isolated from Symptomatic Cases from Andhra Pradesh, India."

https://doi.org/10.5281/zenodo.8046107

Supplementary Table S1. Master data of the study representing variants and their mutations and the number of interactions between the spike and the receptor generated from the molecular dynamic studies performed on each sample nucleotide sequence and the standard ACE2 receptor

Sl. No.	Sample_ID	Collection date	Group	Pango lineage	Clade	Variant	NA_Sub	NA_Del	AA_Sub	AA_Del	Genbank acc no.	Dope score	RMSE	Interactions within spike		Interactions (spike-hACE2)	
														bonded	non-bonded	bonded	non-bonded
1	SVIMS_543985	30.03.2021	G9	B.1.1.7	20I	Alpha	7	9	7	3	ON651722	-411547.719	0.228	212	1821	29	282
2	SVIMS_554429	06.04.2021	G9	B.1.1.7.8	20I	Alpha	8	9	7	3	ON668149	-411547.719	0.228	212	1821	29	282
3	SVIMS_505054	09.03.2021	G58	B.1.1.7.4	20I	Alpha	6	9	6	3	ON668143	-411595.375	0.208	209	1776	12	176
4	SVIMS_35599	28.05.2021	G34	B.1.1.7.8	20I	Alpha	9	9	8	3	ON651697	-411980.344	0.193	225	1837	19	266
5	SVIMS_735971	28.05.2021	G9	B.1.1.7.8	20I	Alpha	8	9	7	3	ON651698	-411547.719	0.228	212	1821	29	282
6	SVIMS_539168	27.03.2021	G9	B.1.1.7	20I	Alpha	7	9	7	3	ON651700	-411547.719	0.228	212	1821	29	282
7	SVIMS_539176	27.03.2021	G9	B.1.1.7	20I	Alpha	8	9	7	3	ON651701	-411547.719	0.228	212	1821	29	282
8	SVIMS_543243	31.03.2021	G9	B.1.1.7	20I	Alpha	7	9	7	3	ON651709	-411547.719	0.228	212	1821	29	282
9	SVIMS_543265	31.03.2021	G42	B.1.1.7	20I	Alpha	6	9	6	3	ON651710	-411691.781	0.212	219	1850	15	251
10	SVIMS_43889	30.03.2021	G9	B.1.1.7.8	20I	Alpha	8	9	7	3	ON651715	-411547.719	0.228	212	1821	29	282
11	SVIMS_543894	30.03.2021	G9	B.1.1.7	20I	Alpha	7	9	7	3	ON651716	-411547.719	0.228	212	1821	29	282
12	SVIMS_543905	30.03.2021	G46	B.1.1.7	20I	Alpha	9	6	10	2	ON651717	-410893.219	0.223	222	1822	19	186
13	SVIMS_46931	02.04.2021	G9	B.1.1.7.8	20I	Alpha	8	9	7	3	ON668134	-411547.719	0.228	212	1821	29	282
14	SVIMS_551012	04.04.2021	G59	B.1.1.7	20I	Alpha	6	9	6	3	ON668144	-412179.312	0.256	206	1853	11	146
15	SVIMS_544029	30.03.2021	G50	B.1.617.2.4	21J	Delta	7	6	8	2	ON668129	-410052.281	0.242	206	1752	10	156
16	SVIMS_543587	31.03.2021	G44	B.1.617.2.35	21A	Delta	9	6	10	2	ON651712	-410459.844	0.23	206	1783	22	205
17	SVIMS_549070	03.04.2021	G57	B.1.617.2.4	21J	Delta	9	6	10	2	ON668142	-410400.125	0.193	220	1804	19	265
18	SVIMS_1444551	20.11.2021	G1	B.1.617.2.4	21J	Delta	11	6	10	2	ON644350	-410901.438	0.193	202	1787	20	297
19	SVIMS_1446548	21.11.2021	G1	B.1.617.2.4	21J	Delta	11	6	10	2	ON644351	-410901.438	0.193	202	1787	20	297
20	SVIMS_1468869	03.12.2021	G1	B.1.617.2.4	21J	Delta	11	6	10	2	ON644352	-410901.438	0.193	202	1787	20	297

Supplementary Table S1. Master data of the study representing variants and their mutations and the number of interactions between the spike and the receptor generated from the molecular dynamic studies performed on each sample nucleotide sequence and the standard ACE2 receptor

Sl. No.	Sample_ID	Collection date	Group	Pango lineage	Clade	Variant	NA_Sub	NA_Del	AA_Sub	AA_Del	Genbank acc no.	Dope score	RMSE	Interactions within spike		Interactions (spike-hACE2)	
														bonded	non-bonded	bonded	non-bonded
21	SVIMS_1486179	10.12.2021	G12	B.1.617.2.4	21J	Delta	10	6	10	2	ON644353	-408948.938	0.264	212	1830	8	123
22	SVIMS_1500968	16.12.2021	G15	B.1.617.2.4	21J	Delta	10	6	9	2	ON644358	-410296.75	0.179	203	1797	22	263
23	SVIMS_1507657	18.12.2021	G13	B.1.617.2.4	21J	Delta	10	6	10	2	ON644354	-411319.312	0.209	211	1766	12	226
24	SVIMS_1508528	18.12.2021	G14	B.1.617.2.4	21J	Delta	13	6	11	2	ON644355	-409678.438	0.205	204	1825	17	192
25	SVIMS_1508534	18.12.2021	G15	B.1.617.2.4	21J	Delta	10	6	9	2	ON644356	-410296.75	0.179	203	1797	22	263
26	SVIMS_1509454	20.12.2021	G16	B.1.617.2.4	21J	Delta	9	6	10	2	ON644357	-409594.438	0.244	217	1752	31	233
27	SVIMS_1534704	29.12.2021	G17	B.1.617.2.4	21J	Delta	10	6	10	2	ON644359	-409987.75	0.179	217	1752	31	233
28	SVIMS_1553168	05.01.2022	G21	B.1.617.2.4	21J	Delta	9	9	10	3	ON644366	-410146.875	0.22	221	1858	23	250
29	SVIMS_735516	28.05.2021	G32	B.1.617.2.4	21J	Delta	11	6	12	2	ON651695	-410369.719	0.19	219	1816	18	203
30	SVIMS_735522	28.05.2021	G33	B.1.617.2.44	21J	Delta	9	6	9	2	ON651696	-409283.031	0.199	210	1743	21	278
31	SVIMS_736318	28.05.2021	G35	B.1.617.2.27	21I	Delta	8	6	9	2	ON651699	-409462.781	0.247	204	1737	25	296
32	SVIMS_243059	05.11.2020	G39	B.1.617.2	21A	Delta	8	6	9	2	ON651706	-410077.312	0.207	213	1888	16	291
33	SVIMS_543502	31.03.2021	G43	B.1.617.2.35	21A	Delta	9	6	10	2	ON651711	-409898.562	0.209	229	1872	15	227
34	SVIMS_543762	31.03.2021	G45	B.1.617.2	21A	Delta	11	6	12	2	ON651714	-410329.25	0.179	203	1715	43	423
35	SVIMS_543938	30.03.2021	G3	B.1.617.2.27	21I	Delta	8	6	9	2	ON651718	-411474.344	0.239	210	1777	17	171
36	SVIMS_543940	30.03.2021	G2	B.1.617.2.44	21J	Delta	7	6	8	2	ON651719	-410636.062	0.195	222	1898	17	183
37	SVIMS_543953	30.03.2021	G39	B.1.617.2	21A	Delta	8	6	9	2	ON651720	-410077.312	0.207	213	1888	16	291
38	SVIMS_543964	30.03.2021	G47	B.1.617.2.99.2	21J	Delta	7	0	7	0	ON651721	-411122.125	0.228	224	1878	14	159
39	SVIMS_543993	30.03.2021	G48	B.1.617.2.99.2	21J	Delta	5	9	5	3	ON651723	-410278.25	0.236	205	1784	9	221
40	SVIMS_544018	30.03.2021	G39	B.1.617.2	21A	Delta	8	6	9	2	ON668127	-410077.312	0.207	213	1888	16	291

Supplementary Table S1. Master data of the study representing variants and their mutations and the number of interactions between the spike and the receptor generated from the molecular dynamic studies performed on each sample nucleotide sequence and the standard ACE2 receptor

Sl. No.	Sample_ID	Collection date	Group	Pango lineage	Clade	Variant	NA_Sub	NA_Del	AA_Sub	AA_Del	Genbank acc no.	Dope score	RMSE	Interactions within spike		Interactions (spike-hACE2)	
														bonded	non-bonded	bonded	non-bonded
41	SVIMS_544061	31.03.2021	G39	B.1.617.2	21A	Delta	8	6	9	2	ON668130	-410077.312	0.207	213	1888	16	291
42	SVIMS_549013	03.04.2021	G39	B.1.617.2	21A	Delta	9	6	9	2	ON668136	-410077.312	0.207	213	1888	16	291
43	SVIMS_549022	03.04.2021	G2	B.1.617.2.44	21J	Delta	7	6	8	2	ON668138	-410636.062	0.195	222	1898	17	183
44	SVIMS_549026	03.04.2021	G4	B.1.617.2	21A	Delta	9	6	10	2	ON668139	-410642.219	0.228	220	1868	20	260
45	SVIMS_549068	03.04.2021	G56	B.1.617.2.44	21J	Delta	8	6	9	2	ON668141	-410003.219	0.219	216	1812	16	182
46	SVIMS_551534	04.04.2021	G60	B.1.617.2	21A	Delta	10	6	11	2	ON668145	-410252.281	0.252	205	1802	12	160
47	SVIMS_552567	05.04.2021	G61	B.1.617.2	21A	Delta	12	7	12	3	ON668146	-409717.844	0.233	214	1802	14	173
48	SVIMS_554417	06.04.2021	G51	B.1.617.2.44	21J	Delta	8	0	7	0	ON668148	-411427.312	0.2	230	1838	32	231
49	SVIMS_664025	12.05.2021	G4	B.1.617.2	21A	Delta	9	6	10	2	ON668152	-410642.219	0.228	220	1868	20	260
50	SVIMS_664027	12.05.2021	G3	B.1.617.2.27	21I	Delta	8	6	9	2	ON668153	-411474.344	0.239	210	1777	17	171
51	SVIMS_664069	12.05.2021	G1	B.1.617.2.4	21J	Delta	11	6	10	2	ON668154	-410901.438	0.193	202	1787	20	297
52	SVIMS_1497945	15.12.2021	G65	B.1.617.2.4	21J	Delta	10	6	10	2	ON668157	-410410.875	0.252	207	1822	16	223
53	SVIMS_539383	27.03.2021	G36	B.1.617.1	21B	Kappa	9	0	8	0	ON651702	-411562.406	0.222	222	1843	18	279
54	SVIMS_539420	27.03.2021	G38	B.1.617.1	21B	Kappa	13	0	10	0	ON651704	-416234.688	0.234	210	1864	12	179
55	SVIMS_544020	30.03.2021	G7	B.1.617.1	21B	Kappa	10	0	8	0	ON668128	-411933.812	0.205	210	1768	11	189
56	SVIMS_44575	01.04.2021	G52	B.1.617.1	21B	Kappa	10	0	9	0	ON668131	-411200.156	0.187	211	1860	16	219
57	SVIMS_46696	01.04.2021	G53	B.1.617.1	21B	Kappa	10	0	9	0	ON668133	-411386.219	0.188	210	1795	11	178
58	SVIMS_547642	02.04.2021	G7	B.1.617.1	21B	Kappa	9	0	8	0	ON668135	-411933.812	0.205	210	1768	11	189
59	SVIMS_549020	03.04.2021	G54	B.1.617.1	21B	Kappa	9	0	8	0	ON668137	-411777.219	0.189	203	1785	22	218
60	SVIMS_554410	06.04.2021	G7	B.1.617.1	21B	Kappa	9	0	8	0	ON668147	-411933.812	0.205	210	1768	11	189

Supplementary Table S1. Master data of the study representing variants and their mutations and the number of interactions between the spike and the receptor generated from the molecular dynamic studies performed on each sample nucleotide sequence and the standard ACE2 receptor

Sl. No.	Sample_ID	Collection date	Group	Pango lineage	Clade	Variant	NA_Sub	NA_Del	AA_Sub	AA_Del	Genbank acc no.	Dope score	RMSE	Interactions within spike		Interactions (spike-hACE2)	
														bonded	non-bonded	bonded	non-bonded
61	SVIMS_1595310	22.01.2022	G6	BA.2	21L	Omicron	28	9	28	3	ON668156	-413835.125	0.242	204	1843	36	375
62	SVIMS_1538474	30.12.2021	G6	BA.2	21L	Omicron	28	9	28	3	ON644360	-413835.125	0.242	204	1843	36	375
63	SVIMS_1538475	30.12.2021	G6	BA.2	21L	Omicron	28	9	28	3	ON644361	-413835.125	0.242	204	1843	36	375
64	SVIMS_1540933	31.12.2021	G6	BA.2	21L	Omicron	29	9	28	3	ON644362	-413835.125	0.242	204	1843	36	375
65	SVIMS_1541972	01.01.2022	G19	BA.2	21L	Omicron	27	9	27	3	ON644364	-412805.906	0.244	194	1824	9	143
66	SVIMS_1544626	03.01.2022	G20	BA.2	21L	Omicron	26	9	26	3	ON644365	-412622.594	0.291	208	1738	27	312
67	SVIMS_1553815	05.01.2022	G22	BA.2	21L	Omicron	29	9	29	3	ON644367	-412623.594	0.254	217	1877	39	356
68	SVIMS_1553846	05.01.2022	G11	BA.2	21L	Omicron	27	9	27	3	ON644368	-412443.656	0.275	199	1797	11	166
69	SVIMS_1553939	05.01.2022	G6	BA.2	21L	Omicron	28	9	28	3	ON644369	-413835.125	0.242	204	1843	36	375
70	SVIMS_1554002	05.01.2022	G23	BA.2	21L	Omicron	29	9	29	3	ON644370	-412792.938	0.274	210	1817	17	194
71	SVIMS_1554016	05.01.2022	G24	BA.2	21L	Omicron	27	9	27	3	ON644371	-412558.25	0.252	205	1784	9	221
72	SVIMS_1565444	08.01.2022	G5	BA.2.10.1	21L	Omicron	29	9	29	3	ON644373	-414187.125	0.292	197	1806	24	328
73	SVIMS_1566269	09.01.2022	G26	BA.2	21L	Omicron	25	9	25	3	ON644374	-412198.844	0.262	208	1812	29	257
74	SVIMS_1567457	10.01.2022	G27	BA.1.1.16	21K	Omicron	20	15	19	5	ON644375	-411964.812	0.196	207	1804	19	275
75	SVIMS_1599159	22.01.2022	G6	BA.2	21L	Omicron	28	9	28	3	ON644376	-413835.125	0.242	204	1843	36	375
76	SVIMS_1602781	28.01.2022	G29	BA.2	21L	Omicron	25	9	25	3	ON644378	-412126.938	0.282	217	1816	34	298
77	SVIMS_1602786	28.01.2022	G6	BA.2	21L	Omicron	28	9	28	3	ON644379	-413835.125	0.242	204	1843	36	375
78	SVIMS_1602788	28.01.2022	G6	BA.2	21L	Omicron	28	9	28	3	ON644380	-413835.125	0.242	204	1843	36	375
79	SVIMS_1602814	28.01.2022	G5	BA.2.10.1	21L	Omicron	29	9	29	3	ON644381	-414187.125	0.292	197	1806	24	328
80	SVIMS_1602852	28.01.2022	G5	BA.2.10.1	21L	Omicron	29	9	29	3	ON644382	-414187.125	0.292	197	1806	24	328

Supplementary Table S1. Master data of the study representing variants and their mutations and the number of interactions between the spike and the receptor generated from the molecular dynamic studies performed on each sample nucleotide sequence and the standard ACE2 receptor

Sl. No.	Sample_ID	Collection date	Group	Pango lineage	Clade	Variant	NA_Sub	NA_Del	AA_Sub	AA_Del	Genbank acc no.	Dope score	RMSE	Interactions within spike		Interactions (spike-hACE2)	
														bonded	non-bonded	bonded	non-bonded
81	SVIMS_1602853	28.01.2022	G30	BA.2	21L	Omicron	28	9	28	3	ON644383	-412465.656	0.239	185	1769	15	285
82	SVIMS_1605030	01.02.2022	G6	BA.2	21L	Omicron	28	9	28	3	ON644384	-413835.125	0.242	204	1843	36	375
83	SVIMS_1607478	03.02.2022	G11	BA.2	21L	Omicron	27	9	27	3	ON651690	-412443.656	0.275	199	1797	11	166
84	SVIMS_1616911	24.02.2022	G6	BA.2	21L	Omicron	28	9	28	3	ON651691	-413835.125	0.242	204	1843	36	375
85	SVIMS_1616944	24.02.2022	G8	BA.2	21L	Omicron	29	9	29	3	ON651692	-412888.625	0.237	212	1825	23	295
86	SVIMS_17514	25.02.2022	G8	BA.2	21L	Omicron	29	9	29	3	ON651693	-412888.625	0.237	212	1825	23	295
87	SVIMS_1623722	29.02.2022	G31	BA.2.56	21L	Omicron	29	9	29	3	ON651694	-412066.906	0.251	206	1793	17	320
88	SVIMS_543592	31.03.2021	G6	BA.2	21L	Omicron	27	9	28	3	ON651713	-413835.125	0.242	204	1843	36	375
89	SVIMS_1599154	22.01.2022	G6	BA.2	21L	Omicron	28	9	28	3	ON668158	-413835.125	0.242	204	1843	36	375
90	SVIMS_1599160	22.01.2022	G66	BA.2	21L	Omicron	27	9	26	3	ON668159	-412772.375	0.238	219	1816	18	203
91	SVIMS_1599206	22.01.2022	G6	BA.2	21L	Omicron	28	9	28	3	ON668160	-413835.125	0.242	204	1843	36	375
92	SVIMS_539385	27.03.2021	G37	B.1.617	20A	unnamed	5	9	5	3	ON651703	-411292.969	0.225	226	1846	16	228
93	SVIMS_1606063	01.02.2022	G63	XU	RC	RC	23	9	23	3	ON668151	-412294.5	0.293	212	1860	19	307
94	SVIMS_1608329	04.02.2022	G64	XU	RC	RC	17	0	16	0	ON668155	-413039.125	0.236	224	1853	17	225
95	SVIMS_1541235	31.12.2021	G18	B.1	20A	unnamed	8	9	8	3	ON644363	-411003.469	0.271	210	1787	20	234
96	SVIMS_1562054	07.01.2022	G25	B.1.551	20A	unnamed	5	15	6	5	ON644372	-410463.375	0.237	209	1775	11	182
97	SVIMS_242746	05.11.2020	G10	B.1.36	20A	unnamed	3	0	2	0	ON651705	-413198.594	0.204	216	1760	15	201
98	SVIMS_543113	29.03.2021	G40	B.1.551	20A	unnamed	6	9	6	3	ON651707	-410407.812	0.204	216	1838	11	220
99	SVIMS_543983	30.03.2021	G41	B.1.36	20A	unnamed	2	9	1	3	ON651708	-411628.906	0.195	189	1726	21	247
100	SVIMS_544017	30.03.2021	G49	B.1.617	20A	unnamed	4	0	4	0	ON651724	-411191	0.232	204	1853	14	261

Supplementary Table S1. Master data of the study representing variants and their mutations and the number of interactions between the spike and the receptor generated from the molecular dynamic studies performed on each sample nucleotide sequence and the standard ACE2 receptor

Sl. No.	Sample_ID	Collection date	Group	Pango lineage	Clade	Variant	NA_ Sub	NA_ Del	AA_ Sub	AA_ Del	Genbank acc no.	Dope score	RMSE	Interactions within spike		Interactions (spike-hACE2)	
														bonded	non-bonded	bonded	non-bonded
101	SVIMS_546609	01.04.2021	G10	B.1.36	20A	unnamed	3	0	2	0	ON668132	-413198.594	0.204	216	1760	15	201
102	SVIMS_549063	03.04.2021	G55	B.1.1	20B	unnamed	6	9	6	3	ON668140	-411089.031	0.211	216	1823	20	245
103	SVIMS_554463	06.04.2021	G62	B.1	20A	unnamed	14	9	14	3	ON668150	-411192	0.254	219	1816	18	203
104	SVIMS_1599935	23.01.2022	G28	B.1.617	20A	unnamed	5	9	4	3	ON644377	-411467	0.182	195	1771	14	198
Group; similar sequences (amino acid sequence) were grouped sequence similarity S1508528 had 6 nucleotide, one AA insertions; S52567 had one Frame Shifts mutation																	

Supplementary Table S2. Comparison of the frequency and percentage of each amino acid substitution reported in the study with the local, national, and global occurrence of the same mutation

SL.NO	AA Mutation	Mutation ratio*	Number of mutations reported (percentage)			
			Present study	Andhra Pradesh	INDIA	Global
1	S:L5F	0.57	1(0.96)	130(1.122)	2669(0.913)	268094(1.701)
2	S:Q14H	15.44	1(0.962)	17(0.147)	193(0.066)	9816(0.062)
3	S:L18F	0.58	1(0.962)	33(0.285)	1200(0.41)	263403(1.671)
4	S:T19R	0.84	25(24.038)	5149(44.422)	93957(32.135)	4517977(28.658)
5	S:T19I	0.50	18(17.308)	2836(24.467)	109478(37.443)	5505540(34.922)
6	S:A27S	0.53	19(18.269)	45(0.388)	74423(25.454)	5407981(34.303)
7	S:H49Y	7.77	1(0.962)	16(0.138)	430(0.147)	19503(0.124)
8	S:A67V	0.12	2(1.923)	46(0.397)	12570(4.299)	2549642(16.173)
9	S:K77T	75.32	8(7.692)	83(0.716)	1592(0.544)	16100(0.102)
10	S:T95I	0.60	17(16.346)	1963(16.936)	43125(14.749)	4290142(27.213)
11	S:G142D	0.69	50(48.077)	3992(34.441)	148735(50.87)	10960428(69.523)
12	S:Y145D	0.12	2(1.923)	12(0.104)	6948(2.376)	2436966(15.458)
13	S:H146Y	19.74	1(0.962)	0(0)	96(0.033)	7678(0.049)
14	S:E154K	129.47	6(5.769)	40(0.345)	4126(1.411)	7025(0.045)
15	S:R158G	125.43	24(23.077)	1(0.009)	903(0.309)	29006(0.184)
16	S:V213G	43.47	20(19.231)	2917(25.166)	93131(31.852)	69740(0.442)
17	S:A222V	0.47	2(1.923)	285(2.459)	10468(3.58)	650702(4.127)
18	S:A262S	4.50	1(0.962)	5(0.043)	537(0.184)	33696(0.214)
19	S:A263P	1378.07	1(0.962)	0(0)	8(0.003)	110(0.001)
20	S:G339D	0.36	17(16.346)	3424(29.54)	103769(35.491)	7234710(45.891)
21	S:S371F	0.45	16(15.385)	2633(22.716)	109783(37.548)	5400421(34.256)
22	S:S373P	0.33	17(16.346)	2761(23.82)	119438(40.85)	7713393(48.927)
23	S:S375F	0.34	17(16.346)	2708(23.363)	117466(40.175)	7689189(48.773)
24	S:T376A	0.48	17(16.346)	2547(21.974)	107872(36.894)	5403417(34.275)
25	S:V382L	43.08	1(0.962)	5(0.043)	633(0.216)	3519(0.022)
26	S:D405N	0.44	16(15.385)	3298(28.453)	113776(38.913)	5477614(34.745)
27	S:R408S	0.46	16(15.385)	3287(28.358)	100248(34.287)	5221564(33.121)
28	S:K417N	0.35	16(15.385)	3256(28.091)	106024(36.262)	7017467(44.513)
29	S:N440K	0.37	17(16.346)	2728(23.536)	90547(30.969)	6880206(43.642)
30	S:K444R	5.69	1(0.962)	12(0.104)	448(0.153)	26643(0.169)
31	S:L452M	4.30	1(0.962)	26(0.224)	1527(0.522)	35293(0.224)
32	S:L452R	0.74	35(33.654)	5954(51.367)	102166(34.943)	7144229(45.317)
33	S:S477N	0.35	18(17.308)	2676(23.087)	96195(32.9)	7780450(49.352)
34	S:T478K	0.54	43(41.346)	8367(72.185)	178664(61.106)	12165046(77.164)
35	S:N481K	86.03	1(0.962)	0(0)	25(0.009)	1762(0.011)
36	S:E484A	0.39	20(19.231)	2768(23.881)	95381(32.622)	7698745(48.834)
37	S:E484Q	59.44	10(9.615)	423(3.649)	9710(3.321)	25504(0.162)
38	S:Q493R	0.65	19(18.269)	2158(18.618)	58296(19.938)	4439904(28.163)
39	S:G496S	0.07	1(0.962)	104(0.897)	6918(2.366)	2240656(14.213)
40	S:Q498R	0.38	19(18.269)	2271(19.593)	90487(30.948)	7567779(48.003)
41	S:N501Y	0.62	37(35.577)	2452(21.154)	97637(33.394)	9012309(57.166)
42	S:Y505H	0.38	19(18.269)	2774(23.932)	93282(31.904)	7583783(48.105)

Supplementary Table S2. Comparison of the frequency and percentage of each amino acid substitution reported in the study with the local, national, and global occurrence of the same mutation

SL.NO	AA Mutation	Mutation ratio*	Number of mutations reported (percentage)			
			Present study	Andhra Pradesh	INDIA	Global
43	S:V511I	253.07	1(0.962)	0(0)	7(0.002)	599(0.004)
44	S:T547K	0.06	1(0.962)	366(3.158)	14173(4.847)	2598710(16.484)
45	S:A570D	2.08	17(16.346)	66(0.569)	5953(2.036)	1236827(7.845)
46	S:T604N	260.01	1(0.962)	0(0)	7(0.002)	583(0.004)
47	S:D614G	0.73	75(72.115)	11515(99.344)	284642(97.352)	15644346(99.234)
48	S:H655Y	0.32	18(17.308)	3951(34.087)	138317(47.307)	8426910(53.453)
49	S:Q675H	3.02	1(0.962)	9(0.078)	851(0.291)	50220(0.319)
50	S:T676I	48.55	2(1.923)	2(0.017)	75(0.026)	6244(0.04)
51	S:N679K	0.29	16(15.385)	3926(33.871)	137427(47.002)	8261869(52.406)
52	S:P681H	0.59	37(35.577)	4033(34.794)	143935(49.228)	9565862(60.677)
53	S:P681R	1.18	36(34.615)	6266(54.059)	110523(37.801)	4606647(29.221)
54	S:T716I	2.57	21(20.192)	64(0.552)	6047(2.068)	1238315(7.855)
55	S:N764K	0.31	16(15.385)	3847(33.19)	120493(41.211)	7826670(49.646)
56	S:D796Y	0.33	18(17.308)	3836(33.095)	124283(42.507)	8150963(51.703)
57	S:G798D	14.82	1(0.962)	73(0.63)	1857(0.635)	10232(0.065)
58	S:N856K	0.06	1(0.962)	334(2.882)	12998(4.446)	2563941(16.263)
59	S:S929I	78.50	2(1.923)	9(0.078)	152(0.052)	3862(0.024)
60	S:D950N	0.86	25(24.038)	4563(39.367)	84291(28.829)	4381998(27.796)
61	S:Q954H	0.30	16(15.385)	3932(33.923)	128498(43.949)	8099585(51.377)
62	S:N969K	0.30	16(15.385)	3912(33.75)	127504(43.609)	8115982(51.481)
63	S:L981F	0.06	1(0.962)	318(2.744)	12901(4.412)	2565539(16.274)
64	S:S982A	2.02	16(15.385)	62(0.535)	6014(2.057)	1198821(7.604)
65	S:Q1071H	223.37	11(10.577)	148(1.277)	4760(1.628)	7465(0.047)
66	S:H1101D	426.61	9(8.654)	60(0.518)	2320(0.793)	3198(0.02)
67	S:D1118H	2.01	16(15.385)	54(0.466)	6066(2.075)	1207740(7.661)
68	S:D1153Y	15.39	1(0.962)	9(0.078)	709(0.242)	9849(0.062)
69	S:K1245R	170.90	1(0.962)	0(0)	19(0.006)	887(0.006)

AA; amino acid, *Ratio; is an estimated value derived by dividing the percentage of a particular mutation observed in this study (fourth column) with percentage of the same mutation reported globally (last column).

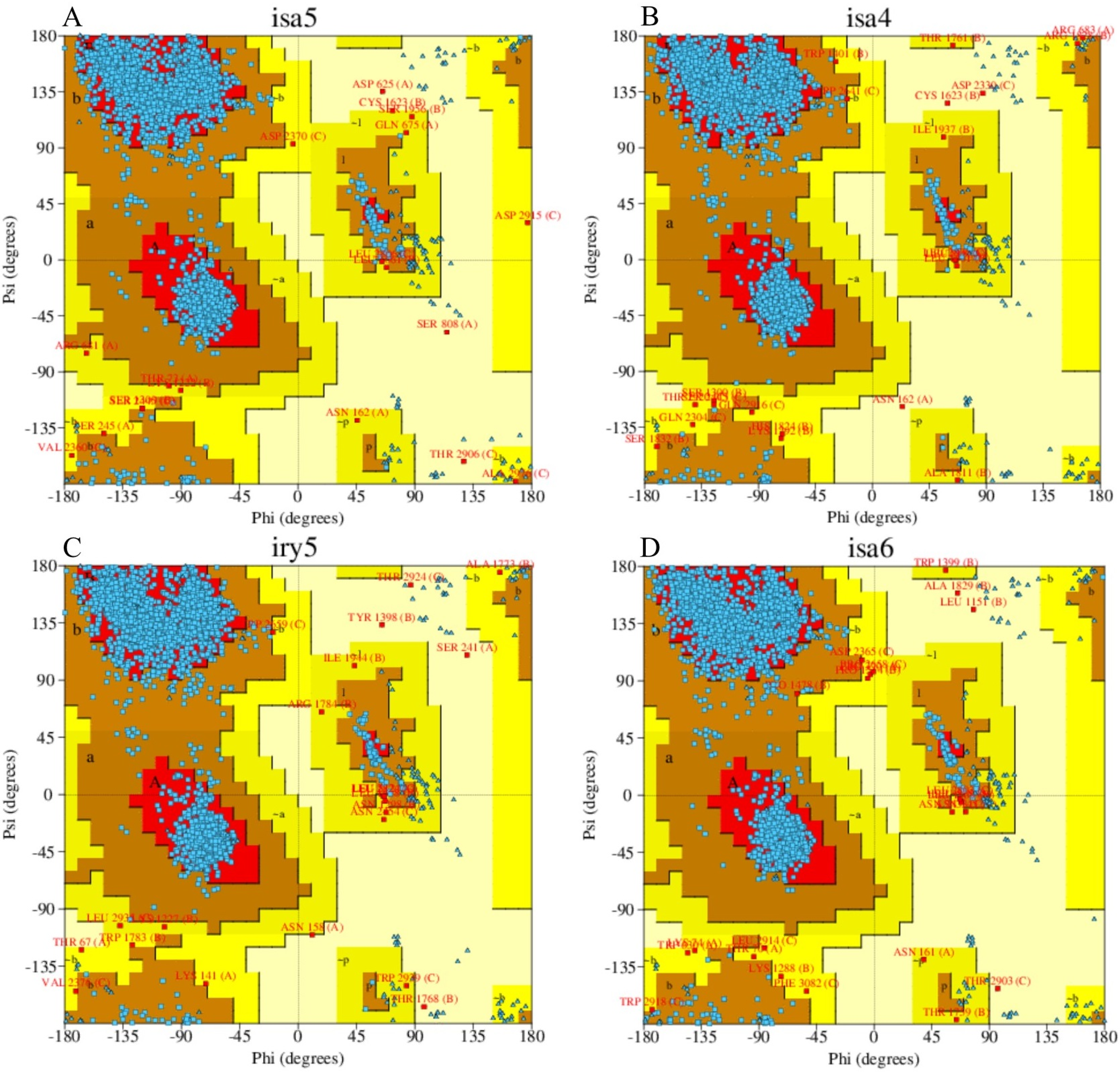

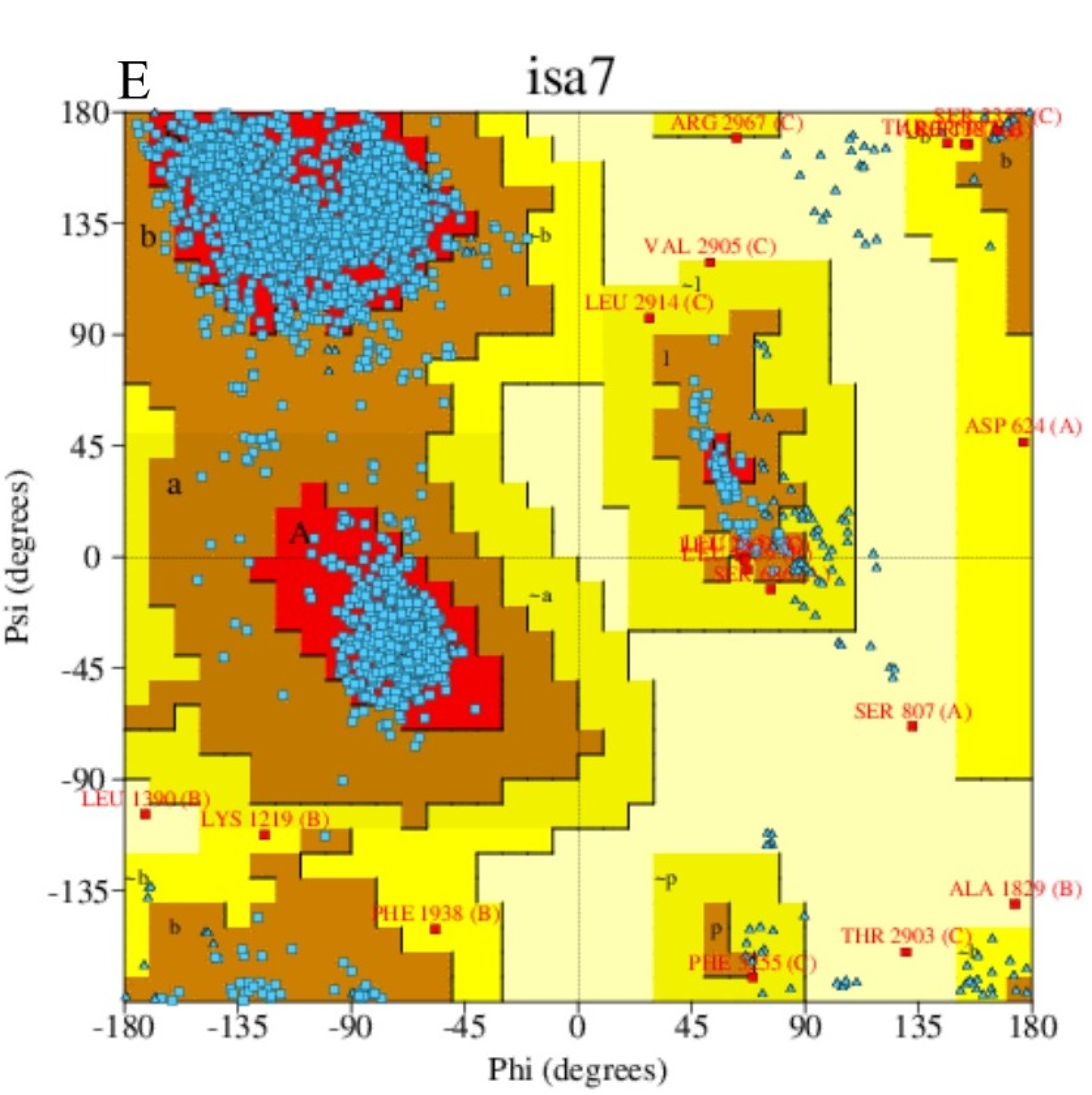

Supplementary Figure S1A-E.

Supplementary Figure S1.

A. Validation of the modeled Alpha variant (sample id 543905) spike protein using the Ramachandran plot of PROCHECK analysis indicated 90.9% (2764), 8.5% (259), 0.5% (16), and 0.1% (3) residues belong to the most favored regions [A, B, and L], additionally allowed regions, generously allowed regions, and disallowed regions, respectively. It is a good-quality model based on more than 90% of the residues that fall in the most favored regions [A, B, and L].

B. Validation of the modeled Delta variant (sample ID 735516) spike protein using the Ramachandran plot of PROCHECK analysis indicated 91.1% (2772), 8.2% (249), 0.6% (18), and 0.1% (3) residues belong to the most favored regions [A, B, and L], additionally allowed regions, generously allowed regions, and disallowed regions, respectively. It is a good-quality model based on more than 90% of the residues that fall in the most favored regions [A, B, and L].

C. Validation of the modeled Kappa variant (sample ID 46696) spike protein using Ramachandran plot of PROCHECK analysis indicated 91.2% (2808), 8.1% (249), 0.5% (16), and 0.2% (5) residues belong to the most favored regions [A, B, and L], additionally allowed regions, generously allowed regions, and disallowed regions, respectively. It is a good-quality model based on more than 90% of the residues that fall in the most favored regions [A, B, and L].

D. Validation of modeled Omicron variant (sample id 1623722) spike protein using Ramachandran plot of PROCHECK analysis indicating 91.0% (2770), 8.4% (256), 0.5% (16), and 0.1% (3) residues belong to the most favored regions [A,B,L], additionally allowed regions, generously allowed regions, and disallowed regions, respectively. It is a good-quality model based on more than 90% of the residues that fall in the most favored regions [A, B, and L].

E. Validation of the modeled unnamed variant (sample id 1599310) spike protein using the Ramachandran plot of PROCHECK analysis indicated 91.2% (2772), 8.2% (248), 0.5% (14), and 0.2% (5) residues belong to the most favored regions [A, B, and L], additionally allowed regions, generously allowed regions, and disallowed regions, respectively. It is a good-quality model based on more than 90% of the residues that fall in the most favored regions [A, B, and L].

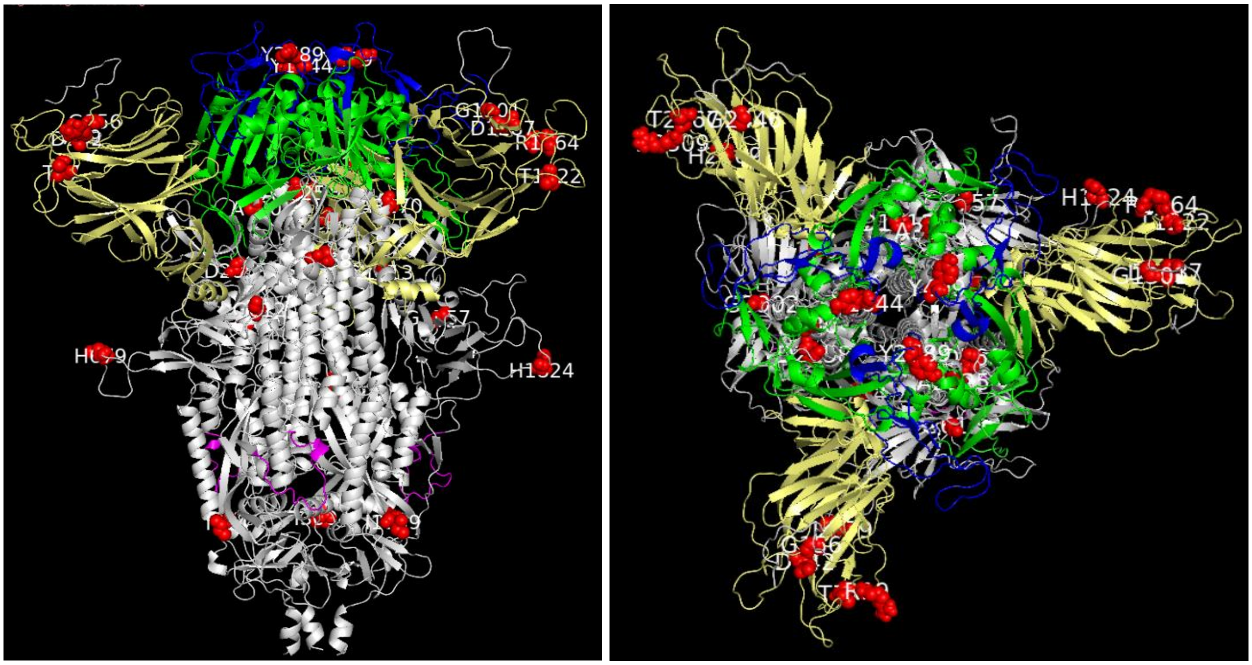

Front view   Top view

Supplementary Figure S2. Schematic of the SARS-CoV-2 spike protein primary structure (trimer) generated from the **Alpha variant (sample ID 543905)** sequence of this study Different domains are shown with different colors: N-terminal domain (NTD): yellow; receptor-binding domain (RBD): green; receptor binding motif (RBM): blue; fusion peptide (FP): magenta; other parts of S1 and S2: silver (gray 90%). Mutations are marked with a red sphere and labeled with the residue name (a single-letter code) and position. Mutations are predominantly observed in the NTD and RBM regions.

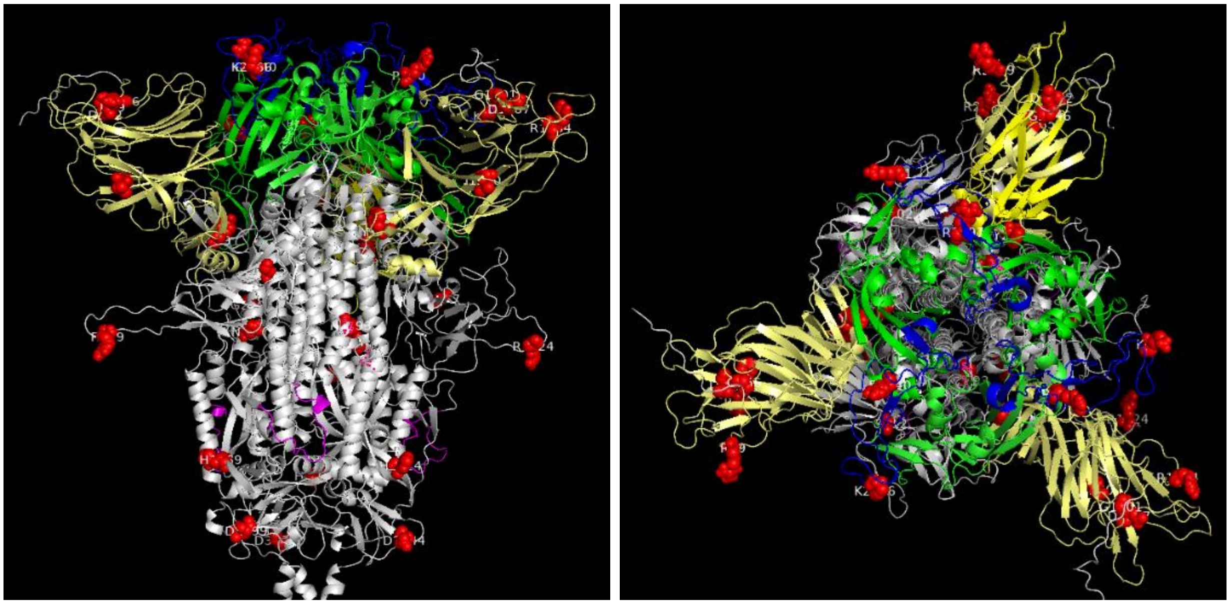

Front view Top view

Supplementary Figure S3. Schematic of the SARS-CoV-2 spike protein primary structure (trimer) generated from the **Delta variant (sample ID 735516)** sequence of this study Different domains are shown with distinct colors: N-terminal domain (NTD): yellow; receptor-binding domain (RBD): green; receptor binding motif (RBM): blue; fusion peptide (FP): magenta; other parts of S1 and S2: silver (gray 90%). Mutations are marked with a red sphere and labeled with the residue name (a single-letter code) and position. Mutations are predominantly observed in the NTD and RBM regions.

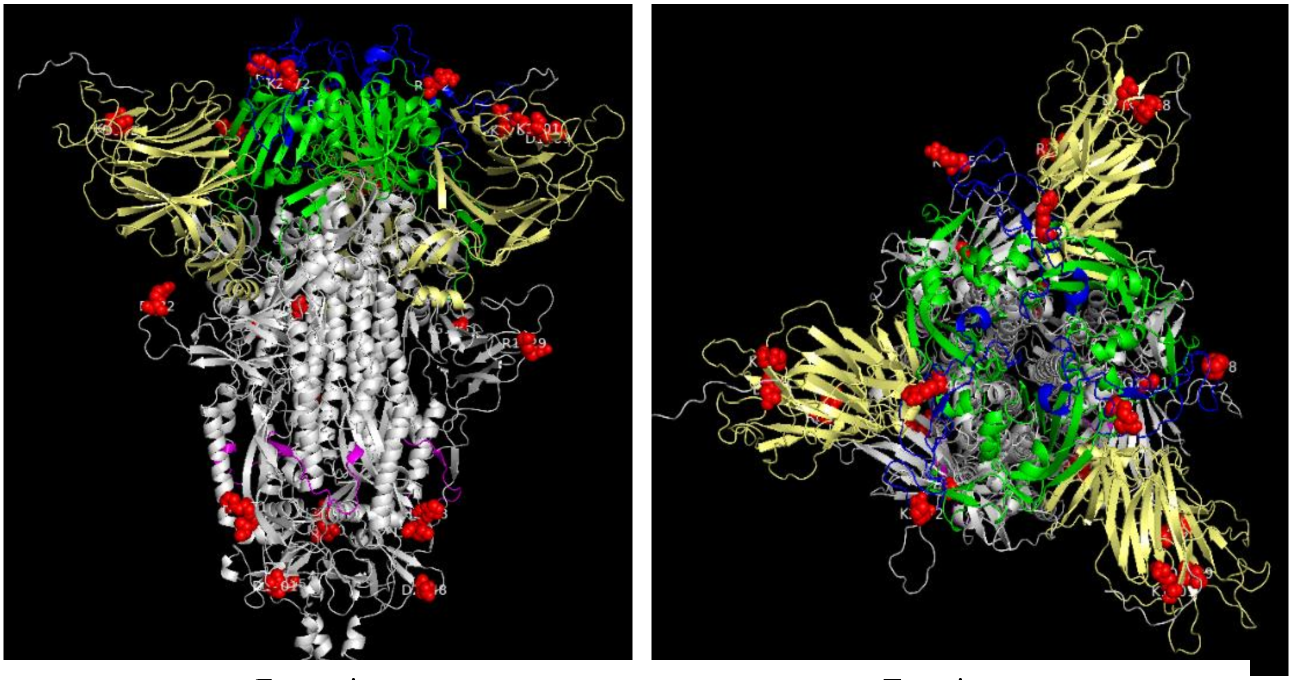

Front view | Top view

Supplementary Figure S4. Schematic of the SARS-CoV-2 spike protein primary structure (trimer) generated from the **Kappa variant (sample ID 46696)** sequence of this study Different domains are shown with distinct colors: N-terminal domain (NTD): yellow; receptor-binding domain (RBD): green; receptor binding motif (RBM): blue; fusion peptide (FP): magenta; other parts of S1 and S2: silver (gray 90%). Mutations are marked with a red sphere and labeled with the residue name (a single-letter code) and position. Mutations are predominantly observed in the NTD and RBM regions.

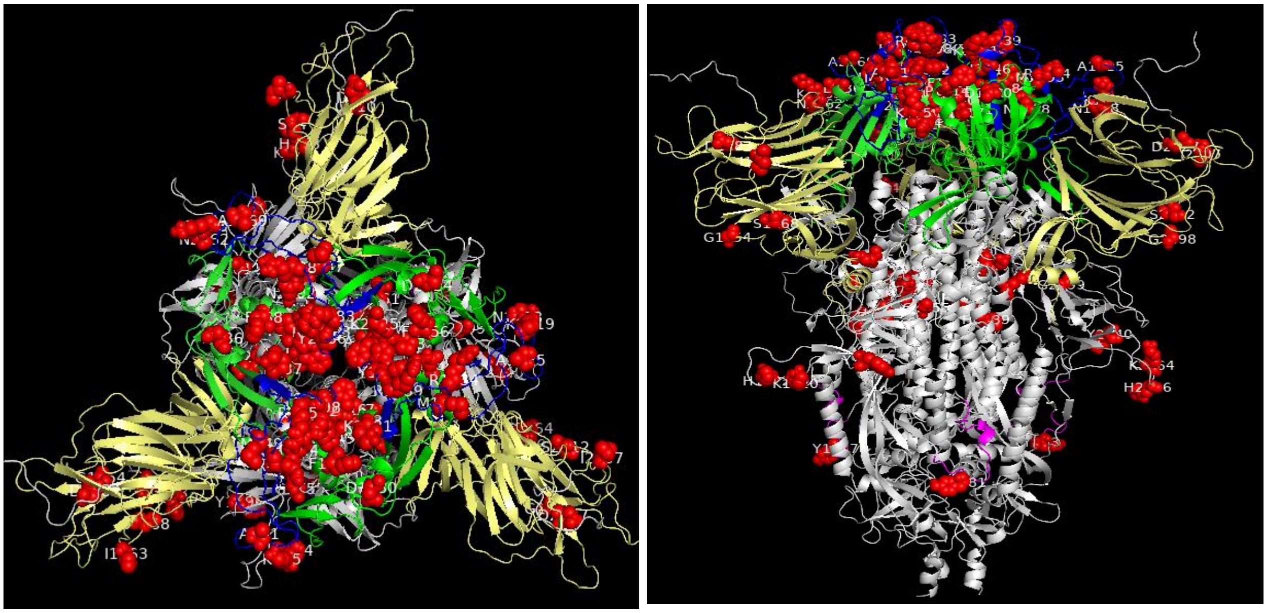

Front view Top view

Supplementary Figure S5. Schematic of the SARS-CoV-2 spike protein primary structure (trimer) generated from the **Omicron variant (sample ID 1623722)** sequence of this study Different domains are shown with distinct colors: N-terminal domain (NTD): yellow; receptor-binding domain (RBD): green; receptor binding motif (RBM): blue; fusion peptide (FP): magenta; other parts of S1 and S2: silver (gray 90%). Mutations are marked with a red sphere and labeled with the residue name (a single-letter code) and position. Mutations are predominantly observed in the NTD and RBM regions.

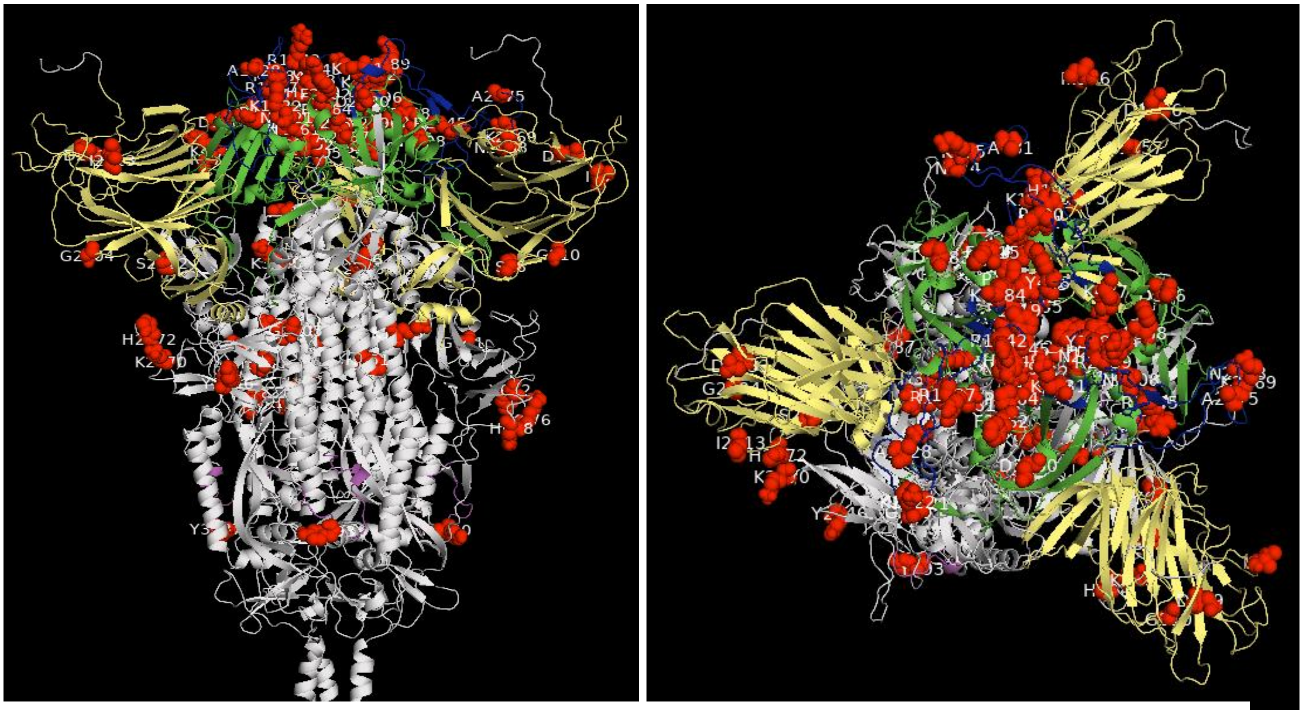

Front view Top view

Supplementary Figure S6. Schematic of the SARS-CoV-2 spike protein primary structure (trimer) generated from the **unnamed variant (sample id 1599310)** sequence of this study Different domains are shown with distinct colors: N-terminal domain (NTD): yellow; receptor-binding domain (RBD): green; receptor binding motif (RBM): blue; fusion peptide (FP): magenta; other parts of S1 and S2: silver (gray 90%). Mutations are marked with a red sphere and labeled with the residue name (a single-letter code) and position. Mutations are predominantly observed in the NTD and RBM regions.

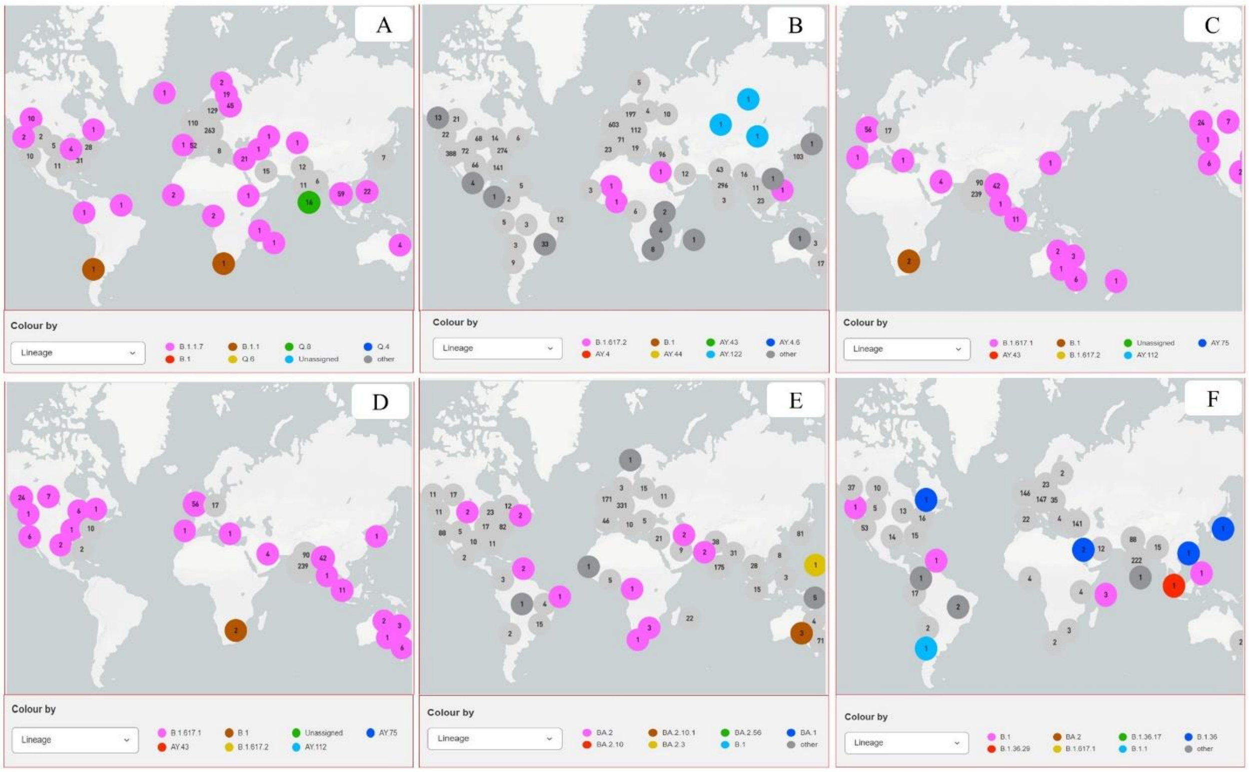

Supplementary Figure S7. Global geographic distribution of the genetic variants of SARS-CoV-2 similar to Alpha (A), Delta (B), Kappa (C and D), Omicron (E), and unnamed (F) variants reported in this study. Source: GISAID (Epicov/Audacity Instant). available at: https://www.epicov.org. Accessed on May 24, 2023.

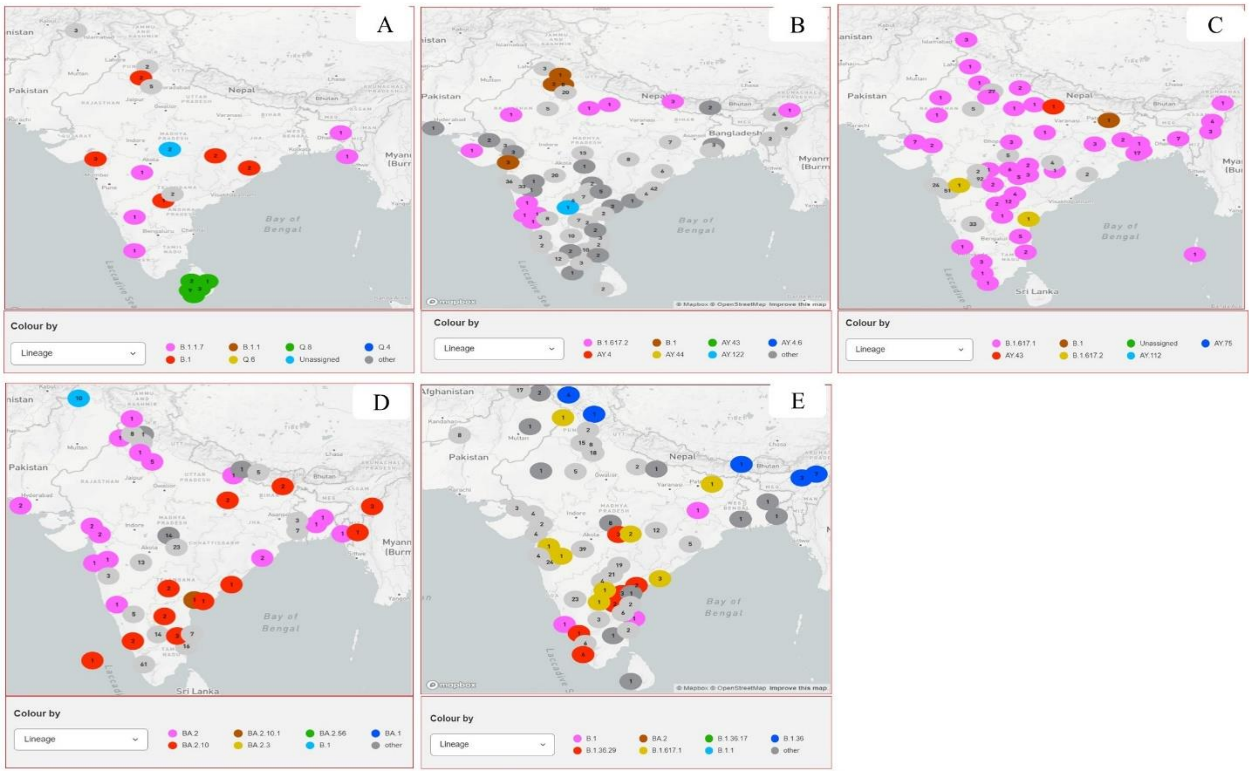

Supplementary Figure S8. Local (India) geographic distribution of the genetic variants of SARS-CoV-2 similar to Alpha (A), Delta (B), Kappa (C), Omicron (D), and unnamed (E) variants reported in this study. Source: GISAID (Epicov/Audacity Instant). available at: https://www.epicov.org. Accessed on May 24, 2023.

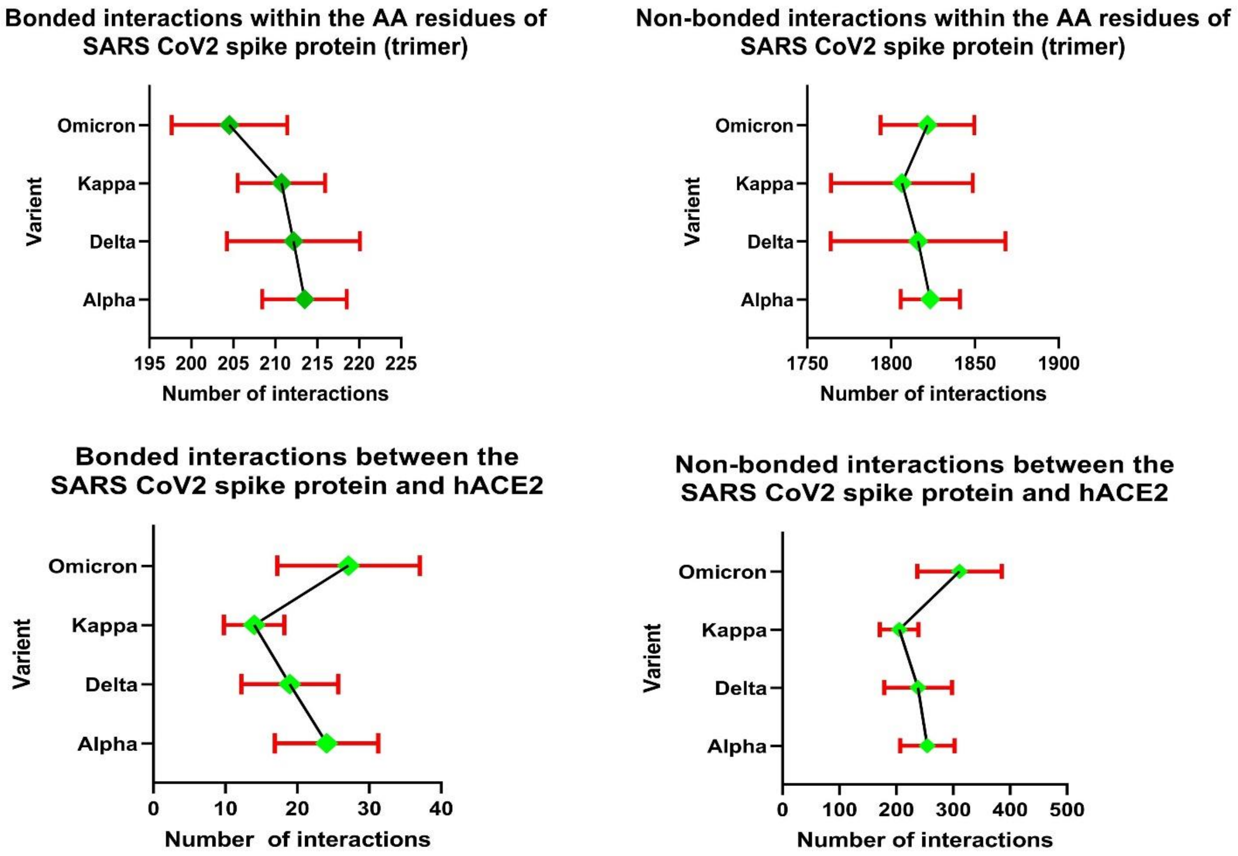

Supplementary Figure S9. Number of bonded and non-bonded interactions observed between the amino acid residues within the spike trimer and spike-ACE2 proteins generated through protein-protein docking studies Different variants observed in the study are reported on the "Y" axis, whereas the number of interactions is indicated on the "X" axis. The rhombus (green) represents the data mean, and the red line indicates the standard deviation. Except for non-bonded interactions within the spike trimer, all other plots showed significant differences.
